# Review of Material Processing Technology for 3D Concrete Printing

**DOI:** 10.3390/ma19030564

**Published:** 2026-01-31

**Authors:** Adam Hutyra, Marcin Maroszek, Magdalena Rudziewicz, Michał Góra, Bożena Tyliszczak

**Affiliations:** Faculty of Materials Engineering and Physics, Cracow University of Technology, Warszawska 24, 31-155 Cracow, Polandmagdalena.rudziewicz@doktorant.pk.edu.pl (M.R.); michal.gora@doktorant.pk.edu.pl (M.G.); bozena.tyliszczak@pk.edu.pl (B.T.)

**Keywords:** 3D concrete printing, 3D concrete mortar, material processing, additive manufacturing, 3D mix formulation

## Abstract

Concrete 3D printing (3DCP) combines materials science with material processing technologies to enable automated, additive construction. This review summarizes findings from the literature and industrial practice on 3DCP mortar formulation with emphasis on the material processing chain. The workflow is examined from raw material storage through handling, mixing, and deposition. The roles of binders, aggregates, dispersed reinforcement, and chemical admixtures are discussed in relation to rheological behavior, buildability, and early-age mechanical performance. The analysis covers storage, dosing, and mixing strategies with respect to mix consistency and overall process reliability, while mortar pumping and extrusion are addressed alongside nozzle-injected additives and automation. Finally, limitations and scalability challenges are outlined with research directions such as continuous mixing, in-line monitoring, and adaptive mix formulation for on-site applications.

## 1. Introduction

Three-dimensional concrete printing (3DCP) brings additive manufacturing (AM) principles to the construction industry. Despite sustained progress, the technology still remains constrained with so-called early deployment stage. Although optimistic in general perspective, recent reviews summarizing market adoption point out a relatively limited number of 3DCP projects worldwide (137 construction projects in Hasani and Dorafshan [1] and 154 reported by Placzek and Schwerdtner [2] during the 2013–2023 period) with activity concentrated mainly within North America (USA) and East Asia (China). Three-dimensional concrete printing offers a high level of design flexibility for structures and optimized material use with waste reduction. Additionally, it addresses sustainability challenges [3,4]. Inheriting features of AM, 3DCP enables the layer-by-layer fabrication of complex concrete structures based on digital models [5], minimizing the need for traditional formwork and manual labor [6]. This approach has earned significant attention in scientific research, with numerous studies exploring aspects such as material deposition (e.g., refs. [7,8]), mechanical properties of printed structures (e.g., refs. [9,10,11]), and development of specialized concrete formulations (e.g., refs. [3,12,13,14]). These works have provided valuable insights into material composition and structural performance. However, comparatively little attention has been devoted to the underlying processing chain [15], despite the fact that raw material handling and mix preparation for pumping and extrusion governs the reliability of 3DCP. While some prior reviews address processing, a consolidated perspective that treats material processing systematically across the full 3DCP workflow remains valuable.

**Figure 1:**
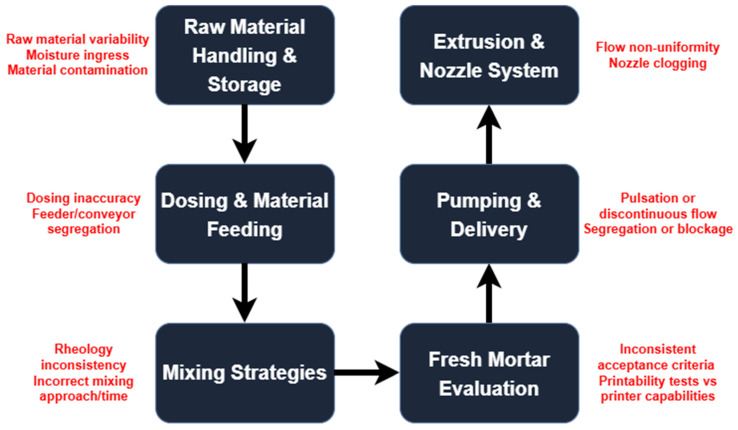
Assumed material processing chain for 3DCP with common failure points.

The preparation of any concrete mix, including the selection and proportioning of raw materials, followed by effective mixing techniques and control of rheological properties, plays a key role in ensuring the final performance of printed structures, similarly to conventional concrete-based construction. However, for 3DCP, issues of extrudability, buildability, or interlayer adhesion of deposited material can originate also directly from applied processing methods. Neglecting these aspects can lead to eventual structural defects or failures and manifest in any of the following stages, affecting possibly the final material curing. Application of 3DCP in the field is hindered by insufficient standardization, acceptance criteria, and limited evaluation methods [16] either for the printing process or the material, often both. This review aims to provide a comprehensive overview of processing technologies utilized throughout every stage of the 3DCP workflow. Therefore, it follows an established scheme found in the majority of existing systems [17], additionally considering early steps such as material handling. Assuming a wider perspective can be advantageous for developing self-contained, complete printing solutions, required for real-world worksites [18,19,20]. As shown in Figure 1, material processing begins with raw materials and storage, follows through mix formulation, and the cycle completes with material transfer and ultimately, deposition. Discussed material processing challenges and potential failure points are put in context of summarized properties of the processed materials that often determine applicable solutions.

## 2. Materials

This section provides the essential background on types and characteristics of basic materials used as ingredients for 3DCP mix formulation. Knowledge of and understanding of the physical properties of raw materials is important for designing applicable processing methods [21]. Importantly, reported properties should be interpreted as indicative rather than directly comparable across studies. Differences arise from measurement methods as well as sample conditioning. Based on various approaches to mix design [14,22,23,24], the following five main material categories are commonly described:

### 2.1. Binding Materials

Binding materials are crucial to 3D concrete printing performance, as they determine the rheological properties of the mortar, directly affect curing time, and ultimately govern mechanical performance. Cementitious materials, particularly Ordinary Portland Cement (OPC, mainly CEM I 52.5R and CEM I 42.5R), dominate in 3DCP due to their established performance and wide availability [25]. Portland cement production is energy-intensive, and the continued reliance on OPC raises concerns regarding sustainability and associated carbon footprint [26,27]. To address this issue, supplementary cementitious materials (SCM(s)) are investigated as partial replacements for OPC [28,29]. Fly ash (FA) is often recommended for its dual role as binder and rheology modifier, improving workability due to its spherical particle morphology. Ground granulated blast furnace slag (GGBFS) is reported to improve strength and reduce autogenous shrinkage after extrusion, as demonstrated by Oh et al. [30]. Both FA and GGBFS are industrial by-products of coal combustion and steel production, respectively. Thus, their use contributes to reduced mix cost and enhanced sustainability. Additional types of SCMs include silica fume (SF), typically dosed at 5–10% of binder mass, and metakaolin (MK), characterized by its extremely fine particle size. Both of which are applied in high-performance printable mortars to enhance pozzolanic reactivity and rheological modification [31]. References for the most commonly applied binders are included in Table 1.

Efforts to reduce OPC content have been documented, with aggregate-to-binder ratios in the range of 1.65–2.15 (corresponding to 419–661 kg/m^3^ of cement) enabling an average 58.2% decrease in OPC use relative to high-cement mixes [36]. From an environmental perspective, although high-strength concretes generally exhibit a greater absolute CO_2_ footprint, they often achieve a lower CO_2_/MPa ratio, thus providing superior environmental efficiency per unit of strength. Moreover, an optimal balance between Ordinary Portland Cement (OPC) and supplementary cementitious materials (SCM(s)) is required, as increased SCM replacement does not always yield improvements in this indicator [37]. Although sustainability benefits are often attributed to 3DCP [27], a more direct life-cycle assessment (LCA) indicates that the environmental advantages of 3DCP are dependent on a specific application rather than inherent to this technology [38].

### 2.2. Aggregates

Fine aggregates, predominantly fine quartz sand [14], constitute the bulk of 3DCP mortars by providing volume and impacting flowability characteristics. Aggregate particle size distribution (PSD) is a decisive factor, as it determines packing density and particle mobility during flow, thereby directly influencing workability [39]. The nominal maximum aggregate size (NMAS) must be carefully controlled to ensure smooth extrusion and minimize possible nozzle clogging. Optimization of PSD improves buildability of deposited layers. Additionally, the inclusion of nanoparticles, due to their high surface-to-volume ratio, can further enhance packing density and rheological properties [40]. However, apart from PSD, the morphology of aggregates can also directly affect mortar extrusion and formulation [41]. Angular type sand particles tend to increase yield stress and consequently the extrusion pressure. Alternatively, round type aggregates can facilitate material flow, often at cost of early strength or buildability. In 3DCP mixtures, aggregate fractions are typically limited to the <1 mm range, as reported by Soares et al. [36] and confirmed in more detailed studies [14,32]. Within this fraction, subdivisions have been distinguished, such as very fine sand with a median particle size of 176.1 μm, medium sand at 498.2 μm, and coarse sand at 840.2 μm [8]. Lightweight aggregates, including expanded perlite and pumice, are occasionally incorporated to adjust density and thermal properties, particularly in non-structural applications.

### 2.3. Recycled Aggregates and Fillers

**Figure 2:**
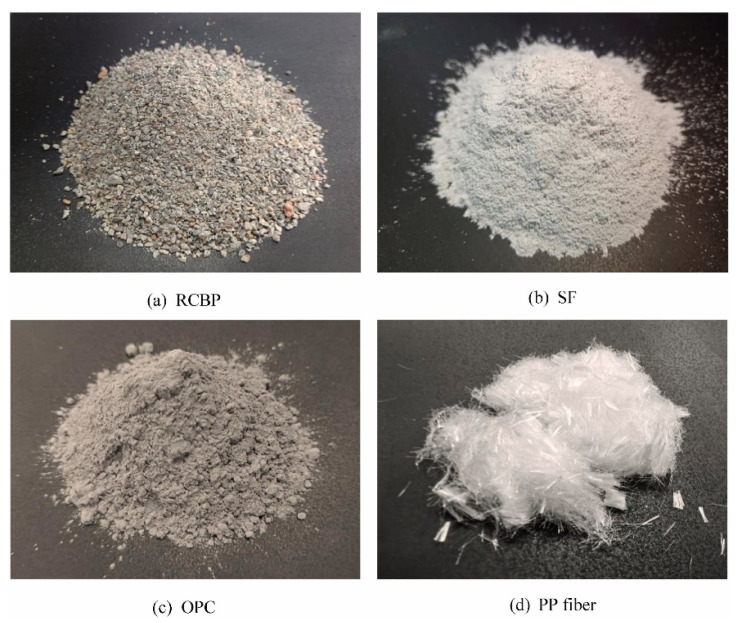
Morphologies of recycled crushed brick powder (RCBP), silica fume (SF), ordinary Portland cement (OPC), and polypropylene (PP) fibers. Adapted from [48].

The inclusion of recycled aggregates (RA) and fillers aligns with sustainability goals [42]. Sourced from construction waste, RA can efficiently replace the function of “volume building” in mix design [21]. Recycled concrete aggregates (RCA), crushed ceramic waste (referred to as Recycled Crushed Brick Powder (RCBP)), and glass cullet [43] serve as eco-friendly substitutes for virgin materials [44]. These alternatives reduce resource extraction and address industrial waste management challenges [45]. Fillers, such as ground glass and recycled plastics, enhance the aesthetic and functional properties of the printed structures. Other fillers provide lightweight mortars with additional unique benefits, as proposed by Rangel et al. [46]. However, their integration requires careful calibration due to variability in properties (as shown in Figure 2), such as particle size and chemical composition [47].

### 2.4. Distributed Reinforcement

Structural reinforcement incorporated during the mixing stage is the common strategy for improving the tensile performance of 3DCP mortars, as summarized by Moelich et al. [49]. Distributing fibers within the mix effectively reduces crack propagation and is compliant with the continuous operation of extrusion systems. Often, this approach eliminates the need for post-print reinforcement installation [26]. The primary objective of fiber incorporation is to compensate for the brittle nature of cementitious materials by enhancing resistance to multiple modes of loading, while also mitigating shrinkage, anisotropy, and weak interlayer bonding through microcrack bridging [40]. In practice, fiber dosages are typically maintained within 0.5–1.5% by volume, balancing mechanical benefits with pumpability and extrusion stability. Among synthetic options, polypropylene (PP) fibers (an average diameter of 40 μm and lengths of 6 or 12 mm) are widely adopted [50], and their inclusion has been shown to increase compressive strength by up to 31% at 28 days [7]. Polyvinyl alcohol (PVA) fibers, with a diameter of approximately 150 μm and length of 12 mm, have also been successfully implemented [51]. Natural fibers, including cellulose, jute, flax, and coconut, are gaining attention for sustainability benefits; however, their geometry requires careful control to avoid nozzle clogging. Islam et al. [52] reported tensile strength improvements of 15–24% for jute-reinforced mortars. Advanced fibers, such as basalt, steel, and carbon [53], have been explored for high-performance applications, though their higher stiffness and density necessitate precise rheological adjustments to maintain printability. In addition to fiber type and dosing, the fiber orientation during pumping and extrusion stages should also be considered. Studies on reinforced 3DCP mortars (e.g., ref. [54]) correlate anisotropy of mechanical properties with fiber orientation.

### 2.5. Admixtures

Admixtures play a crucial role in tailoring the fresh and hardened properties of 3DCP mortars, balancing workability, curing process, and long-term performance. Superplasticizers, most often based on polycarboxylate ether (PCE), enhance flowability and reduce the water-to-cement ratio, thereby improving pumpability, extrusion consistency, and the mechanical properties of printed elements [25,55,56]. Mentioned studies include commercial formulations, mainly MasterGlenium^®^ (BASF, Ludwigshafen, Germany) and ViscoCrete^®^ (Sika Group, Baar, Switzerland). Viscosity-modifying agents (VMA), typically hydroxypropyl methylcellulose (HPMC), dosed at approximately 0.24% of binder mass, are incorporated to improve cohesion and buildability between successive layers [25]. Accelerators, such as calcium chloride (CaCl_2_), promote early strength development. Kazemian et al. [16] demonstrated that additions of 1–3% of binder mass significantly shortened setting times; however, such high doses may violate regulatory limits and impose corrosion risk. For durability enhancement, shrinkage-reducing agents [49] and, occasionally, hydrophobic modifiers are applied to mitigate cracking and improve resistance to environmental exposure. Overall, admixture optimization is essential to achieving the necessary compromise between rheological stability, open time, and final structural performance [57]. From a process control perspective, the impact due to temperature can affect performance of admixtures and result in variability in mortar parameters such as workability or structural build-up. For this reason, admixture handling and dosing should account for expected ambient conditions at the time of mixing and printing as investigated by Xie et al. [58].

## 3. Material Storage and Dosing

### 3.1. Material Management Strategies

Effective management of raw materials is an integral component of any construction process; however, it is often overlooked in the context of 3DCP. Comprehensive strategies must address the entire workflow, from material sourcing to on-site application, ensuring reliable performance and minimizing waste generation. The key steps include accurately determining material requirements based on project specifications, planning delivery schedules to prevent delays, and optimizing storage to account for limited workspace, thus enhancing logistical efficiency [59]. Planning strategies adapted from conventional construction [60] can often be used, particularly for constrained worksites, where space and organizational limitations mandate precise coordination. The integration of technologies, such as Radio-Frequency Identification (RFID) for material identification and tracking, enhances transparency, operational control, and monitoring of material quality and quantity. Maintaining consistent raw material properties for 3DCP is of significant importance, particularly for systems utilizing continuous mixing units, where any fluctuations in material characteristics can disrupt the printing process downstream. This imposes regular quality inspections upon delivery and throughout the workflow. Just-In-Time (JIT) supply chain strategies [61] are well-suited to 3DCP, as they minimize inventory costs and ensure materials meet quality requirements. What is more, advanced tools, such as Value Stream Mapping (VSM) and Geographic Information Systems (GIS), can streamline material flows, reducing inefficiencies in supply chain logistics as described by Raza et al. [62] in on-site 3DCP section. As 3DCP becomes an increasingly standardized technology, the importance of raw material management practices will grow, serving as a foundation for scalable, efficient, and reliable implementation of this additive construction method. Properly managed material supply chains not only improve project timelines and cost-effectiveness but also contribute to sustainability by reducing waste and minimizing the carbon footprint associated with transportation and storage.

### 3.2. Bulk Storage

Dry mix components, such as OPC and various aggregates, are typically delivered in bagged or bulk forms. Cement, due to its properties and significance in the 3DCP process, requires protection against environmental conditions, contamination, and moisture. Bagged cement should be transported in covered vehicles to minimize exposure, while bulk cement is delivered using specialized tankers equipped with pneumatic or gravitational loading and unloading systems. Aggregates are usually transported in bulk with less strict protection requirements; however, preventing contamination and cross-mixing of grades remains essential. Water is often delivered to temporary on-site reservoirs, particularly for short-term projects or locations without reliable water sources, while liquid admixtures are transported in labeled barrels or canisters to ensure traceability [57]. Bagged cement requires isolated, dry storage areas, preferably indoors, with measures such as raised pallets to prevent moisture ingress. Bulk cement is stored in steel silos designed for pneumatic loading, depending on the project scale. These silos often feature conical bottoms with slopes of 55–60 degrees to ensure complete material discharge and prevent residual build-up [63]. Alternatively, horizontal silos, while offering lower capacities, may be preferred for their mobility. Aggregates are generally stored in stockpiles or enclosed bins, with covered storage providing better control over aggregate properties such as moisture content and enabling automated dosing into mixers. According to EN 196-1:2016 [64], commonly used standard sand should not exceed a moisture content of 0.2% by mass, which is determined after drying at 105–110 °C.

In addition to moisture control, temperature is the second major concern for the process, especially for on-site preparation. Elevated temperatures can accelerate early hydration and reduce open time, thus disrupting the printing process.

Notably, many leading 3DCP companies (e.g., Sika Group and XtreeE, Nanterre, France) currently tend to favor pre-mixed, high-performance mortar stored in bags in order to avoid on-site batching. Such a strategy allows for simplification of operations for customers without specialized experience or training. This approach omits the on-site mixing stage, sacrificing the material adaptability to specific printing conditions for the convenience of process workflow. Nevertheless, this tendency should not hinder the development of more advanced 3DCP systems, including raw material processing capabilities.

### 3.3. Feeding

**Figure 3:**
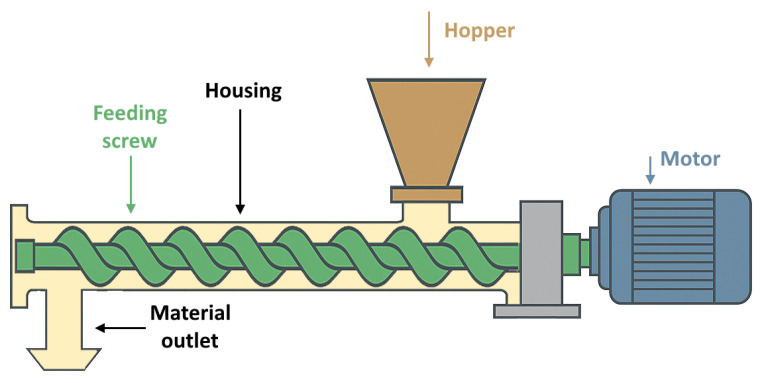
Schematic of a screw auger.

Material feeding into the mixer is the final step in the preparation of dry concrete mixes for 3DCP. The precision and consistency of this stage directly affect the mix composition and, consequently, the quality of the mixed concrete [65]. A common solution for feeding bulk dry materials is the use of screw augers (the principle of operation shown in Figure 3), widely employed across various industries. These devices are adaptable to various granular materials, including cement, aggregates, and solid filler materials [66]. The versatility of screw conveyors originates from adjustable design parameters, such as the length-to-diameter ratio (l/d), screw geometry, and flight angle. According to Minglani et al. [67], optimal pitch-to-diameter ratios are typically around 0.5–1.0, where higher values improve volumetric efficiency but also raise torque demand. The choke length, i.e., the screw section under the hopper, is most effective at about one pitch distance at low speeds and two pitches at higher speeds, while longer choke sections greatly increase torque without improving throughput. Practical designs reported for granular materials use screw diameters of 50–140 mm with l/d ratios of about 20–40, and industrial cement screws extend this up to 40–60 with added hanger bearings. One of the advantages of screw conveyors is their enclosed design, which minimizes dust emissions, while their throughput can be precisely controlled by adjusting the speed of the driving electric motor [68].

Vibratory feeders represent an alternative method for dosing dry materials. They enable accurate feeding through regulation of vibration frequency and mass displacement and are particularly effective for free-flowing powders. However, their performance is sensitive to particle size distribution and material cohesiveness. In particular, for cohesive fine powders, such as cement, flow fluctuations and instabilities have been observed [69]. Despite these limitations, vibratory feeders offer a robust option for controlled dosing when appropriately matched to material characteristics.

On the other hand, belt conveyors are not considered suitable for 3DCP material handling. While they are valued in conventional concrete production for simple construction, long-distance transport, and ease of cleaning, their operation is optimized for large material throughputs rather than precise dosing. Moreover, uneven feeding, susceptibility to segregation, and the difficulty of isolating conveyed material from the environment make belt conveyors less compatible with the requirements of 3DCP applications conducted under construction site conditions.

## 4. Concrete Formulation

In order to correctly introduce and combine all of the materials for 3DCP, a two-stage mixing system is often utilized. The homogeneity, stability, and quality of the fresh concrete mix are directly influenced by the type of mixing employed [70]. These factors significantly affect the extrudability, buildability, and overall performance of printed structures. This section explores the primary mixing approaches and equipment applicable to 3DCP for their strengths and limitations with regard to production scale and material requirements. It is worth noting that the field of mixing systems is still being researched and developed, e.g., ref. [71].

### 4.1. Mixing Types and Equipment

Mixing equipment is a fundamental element of the 3DCP workflow, as it governs material homogeneity and fresh mortar consistency. The choice of mixer is closely linked to production scale and material composition. Planetary mixers are versatile and widely employed for small-scale batches [72], providing thorough blending through combined tool and container rotation, and are therefore common in laboratory testing of new formulations. Paddle mixers are suitable for medium-scale production, while high-shear mixers, despite their higher complexity and cost, offer greater throughput and improved dispersion, particularly effective for additives with challenging rheology such as nanoclay [73]. At the industrial scale, continuous mixers, most notably horizontal twin-shaft mixers, are preferred [70], as they ensure uninterrupted material flow and allow real-time dosing adjustments [35], thereby minimizing delays between mixing and extrusion. In both batch and continuous systems, a two-stage strategy, where dry components are blended prior to addition of water and admixtures, has been shown to enhance dispersion and improve mortar performance [74,75,76,77,78].

Evidence from the interlaboratory study [79], which compared both batch and continuous mixers, indicates that absolute quantitative comparisons are hindered by high variability in materials and equipment. Consequently, research emphasis has shifted toward relative performance of printed versus cast samples, with focus on mechanical properties. Nevertheless, advancing the 3DCP workflow requires extending research beyond laboratory-scale batch mixing to continuous systems equipped with in-line monitoring capabilities. Recent developments in in-line rheology measurement [80] provide a viable pathway for quantifying extrudability during processing, supporting scalability and reliability in large-scale continuous extrusion.

### 4.2. Dry Mix

The preparation of the dry mix ensures uniform distribution of ingredients prior to the addition of water. The primary component is cement, commonly used OPC or partially replaced with SCM(s), which acts as the main binder. Notably, blended cements, including OPC combined with GGBFS, are also utilized to achieve specific performance requirements. Silica fume, a pozzolanic material, is incorporated to enhance both mechanical properties and mix stability. Fine quartz sand covers the majority of the mortar volume and is often added after the initial distribution of lighter materials. Mineral additives such as fly ash and metakaolin are commonly included to modify the mix structural and flow characteristics. Nano-clay is introduced to improve shape retention and rheological control [73]. Adding reinforcing fibers at this stage is generally advised [35,81]. A mixer with high-speed operation is beneficial for even distribution of fiber additives, as those often tend to accumulate in localized entangled groups due to elongated shape. Dry mixing is typically performed for fixed durations, with the initial stage of low speed and a higher intensity final blending, as demonstrated by Baz et al. [75].

### 4.3. Wet Mix

The wet mix is prepared by incorporating water and chemical admixtures into the previously homogenized dry blend of ingredients. Reported formulations for 3DCP mortars most commonly adopt a water-to-binder (w/b) ratio in the range of 0.36–0.41, as summarized by Soares et al. [36]. In this process, a workable mortar is achieved through the activation of the binder [82] and subsequent development of rheological properties [83,84]. This process demands precise dosing and a specific sequence of ingredient addition. The w/b ratio is crucial in achieving the desired consistency of the mixture and must be completed exclusively during this stage, as attempts at regulating mix consistency with additional water volume later in the process will lead to deteriorated mechanical properties. Superplasticizers are employed to enhance the workability of the mix while minimizing the water content, thereby maintaining a low w/b ratio and improving the overall performance of the printed structure. Agents, such as admixtures or corrosion inhibitors, can be included to further adjust the 3DCP mortar for specific applications [18,85]. Mixing times and mixer speeds may vary depending on the type of mixer and the specific properties of the concrete. Controlling wet mix preparation is mandatory for ensuring uniform hydration and consistency within the delivered mortar. For the advanced 3DCP systems (e.g., ref. [86]), a mixing unit is often complementary with the pumping stage for the reduction of material delivery time to an extrusion head of a 3D printer.

## 5. Workflow

The 3DCP process can be described as a sequence of steps between the structural design phase, through material preparation and deposition [85,87], resulting in the final printed structure [20,88,89]. Initially, a digital 3D model of the desired geometry is created, usually by means of Building Information Modeling (BIM) technology, containing information for the entire printing process [90]. Consequently, a suitable concrete mix is to be chosen with properties balanced to the task at hand [91,92]. It is essential for the mix to be reliably transferred throughout all of the stages—pumped, extruded via the printing nozzle, and cured post-deposition. The parameters of a freshly formulated mortar can be maintained within only a limited time after mixing (Kazemian et al. [16]). The next phase involves the transportation of the mix to the print head, typically through a pump. Extrusion is performed by precisely depositing successive layers of concrete to construct a three-dimensional structure. During this process, the nozzle traces wall outlines and internal segments of the assumed geometry, maintaining parameters such as printing speed and layer thickness controlled to ensure the final quality of the printed structure. Once the printing is completed, the concrete undergoes curing, developing strength and resistance. Usually, the ultimate mechanical parameters are achieved after 28 days of curing [10]. This period may be accelerated by applying accelerating admixtures or reducing w/b ratio by adding superplasticizers. Ultimately, the 3DCP process balances the printing-related properties and mix formulation to match the material challenges [22,93], specific structural features, and expected environmental conditions.

### 5.1. Requirements

One of the most fundamental properties of a 3DCP mortar is buildability [47,94], defined as the material’s ability to retain the cross-sectional dimensions of an extruded filament and to withstand the load of subsequent layers without significant deformation [95]. Mortars with optimal buildability combine early stiffness with rapid structural build-up [92,96]. In practical terms, lower deformation values correspond to higher buildability. Simple evaluation methods include printing multiple layers and comparing the achieved height against the theoretical height (product of layer count and layer thickness), provided that the extrusion coefficient has been correctly calibrated to match material flow rate with nozzle size.

Workability, in contrast, reflects the ease and uniformity of placing and finishing concrete, equally relevant for 3DCP and conventional applications. It is associated with material flow under agitation and typically assessed using the slump flow test [89]. Balancing workability and buildability is crucial, since higher flowability facilitates extrusion but compromises structural retention. The yield stress parameter represents the minimum stress needed to initiate flow, and must be low enough to enable extrusion yet high enough to support the deposited layers [97]. Studies have reported printable ranges between 0.5–2.0 kPa [83] or 1.5–2.5 kPa [50] for improved stability. Rapid yield stress development is essential for sustaining loads from stacked layers, with Perrot et al. [74] reporting a rate of approximately 54 Pa/min as satisfying.

Thixotropy, the ability of a material to reduce viscosity under shear and recover it afterwards, is critical for extrusion and rapid stiffening [35,83,98,99,100], and can be enhanced by fiber reinforcement.

The water-to-binder ratio exerts a major influence on rheology and strength; while lower ratios improve buildability and compressive strength, adequate flowability is best achieved through superplasticizers rather than excess water.

Open time, defined as the workable duration of a mortar, is a key factor for continuous extrusion without clogging or deformation. Reported values include approximately 30 min for mixes modified with silica fume (SF) or nanoclay, and 45 min for mortars incorporating viscosity-modifying agents (VMA) [36,50]. The addition of chemical retarders can extend this period further, e.g., to 70 min at a dosage of 0.5% [91]. Formulations with open times below 15 min are generally considered unsuitable for 3DCP, as they do not allow sufficient processing time.

Rheological parameters promoting pumpability often contradict those required for buildability. Thrane et al. [101] reported a plastic viscosity of (38.7 ± 4.5) Pa·s and yield stress of (0.59 ± 0.08) kPa for an OPC–fly ash mortar, whereas substitution with limestone filler yielded lower values of (21.1 ± 2.4) Pa·s and (0.27 ± 0.03) kPa, underlining the sensitivity of rheological response to binder composition.

From a system-design perspective, hose diameters in the range of 20–50 mm have been reported as suitable for 3DCP [39]. The selected diameter must reflect both mix viscosity at the relevant shear rate and pumping capacity, since smaller diameters increase pressure demand. Proper coordination of hose sizing with the nozzle orifice is essential to maintain flow continuity and prevent segregation or blockage.

### 5.2. Mix Evaluation Methods

**Figure 4:**
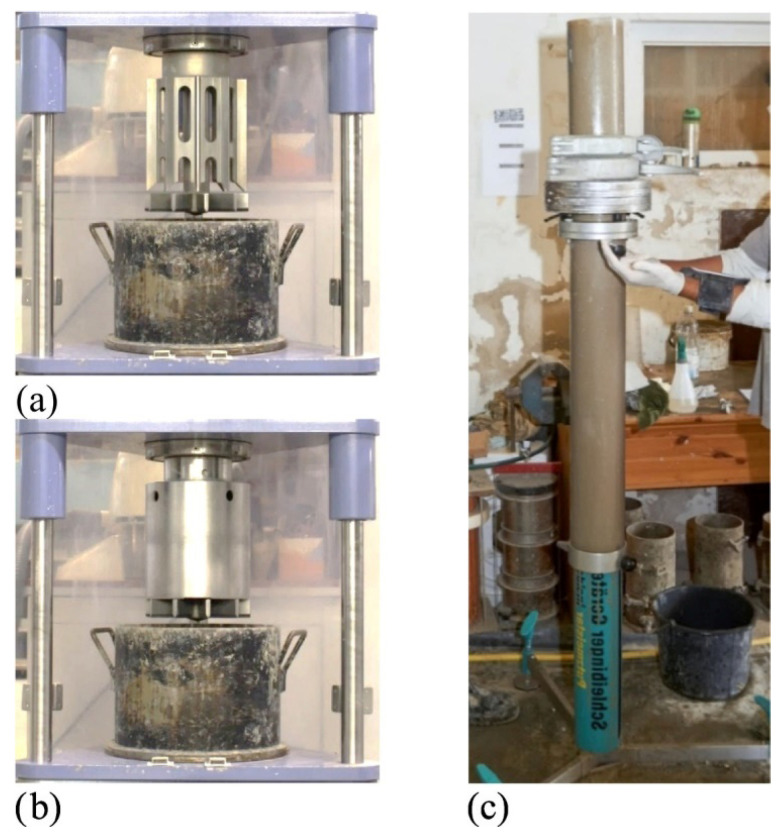
(**a**) Regular rheometer, (**b**) tribometer, (**c**) sliding rheometer presented in ref. [103], after ref. [104].

Ongoing development of cement-based mortars for 3DCP is often hindered by a lack of accepted criteria describing and measuring its properties [102]. The majority of testing approaches are directly adapted from standards for evaluation of concrete in traditional construction field (e.g., rheometers as shown in Figure 4). Although measuring multiple basic parameters appears to be a satisfying approximation, there are some crucial requirements that are not represented in industrial standards. In general, those arise from extrusion-related issues or desired material consistency, outside of values provided by standards. One of the major undefined issues for 3DCP is the lack of standardized procedure for investigating the structural build-up rate after material deposition (under shear cessation, directly impacting buildability and layer bonding [41]). Deficiencies in testing and classification methods are one of the most significant barriers limiting the recognition of 3DCP-based technologies as a valid construction method worldwide.

Table 2 presents the common mortar evaluation methods applicable to 3DCP systems (standardized and proposed in literature).

In summary, the extended control over the mixing process, optimization of mortar performance according to specific challenges of 3DCP, and reliable methods of testing both freshly produced and cured material are key to ensuring quality and technological advancement in additive manufacturing with concrete.

## 6. Material Delivery and Deposition

Effective transfer of freshly mixed material from the mixing unit to the printhead is the next essential stage in 3DCP. The delivery system must maintain mortar consistency while ensuring continuous and stable flow under printer-specific constraints.

### 6.1. Pumping

**Figure 5:**
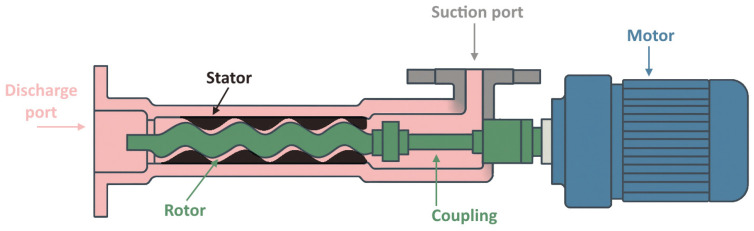
Progressive cavity pump components—schematic.

When considering the transfer of a fresh mortar, it is essential to differentiate between the two stages: firstly, pumping from the mix station to the print head, and secondly, direct material deposition within the actual printer’s workspace. In the first scenario, mortar must be fed through significant lengths of hose. Despite choosing specialized hoses designed for mortar delivery, depending on the rheological parameters of a mortar, significant pressures often must be generated in order to overcome head and other losses [84,103,116]. A well-established solution in the construction industry for such cases is a progressive cavity pump. Characterized by a simple rotor-stator construction (as shown in Figure 5), this type is known for its robustness, ease of cleaning, and maintenance. Since the mixing unit often remains stationary during most of the printing process, relatively high driving torque and the own weight of such a pumping unit can be accepted. For 3DCP, the size (diameter) and geometry of a pump can be adapted from well-established units available for pumping mortars and plaster compounds from the construction industry. However, because printable formulations often contain dispersed fiber reinforcement and require higher yield stress during processing, careful pump selection and modifications are necessary to handle significantly higher pumping loads and general component wear.

Pump performance is dependent on rotor revolution rate; hence, it can be directly controlled by adjusting drive motor speed. Importantly, progressive cavity pumps are suitable for transferring aggregates in sizes practical for 3DCP (up to 3 mm). On the other hand, the use of progressive cavity pumps does not solve issues associated with pumping mortars with long fibers distributed within the volume as it may result in tangling and consequent failure to generate pressure due to a lack of internal sealing. Additionally, any periods of operation with insufficient hydrodynamic lubrication due to mortar presence (essentially, dry runs) before the entire cavity is primed lead to accelerated surface wear and degradation of stator geometry, consequently resulting in sealing issues and final loss of performance. Such events may occur due to improper maintenance and preparation or flawed system design and must be avoided.

Peristaltic pumps are occasionally associated with 3DCP equipment, primarily for dosing applications. Their simple construction, low maintenance requirements, and volumetric operating principle enable accurate delivery of liquids, making them suitable for supplying either water or liquid admixtures and plasticizers. Although one may find large peristaltic pumps employed in the construction industry for conveying mortars, such materials are typically significantly more flowable than 3DCP mixtures. Peristaltic systems inherently produce a pulsating discharge, which necessitates use of the flow-buffering devices. Consequently, peristaltic pumps remain limited to auxiliary roles in 3DCP, while primary material transport generally relies on progressive cavity and screw-type pumps, which provide reliable and more robust operation.

### 6.2. Extrusion

Direct extrusion from the mixing unit onto a printing space is rarely employed in industrial practice, apart from the simplest systems for testing and experimentation. This is mainly due to inertial effects from the distance separating a pumping unit and the given extrusion nozzle. Quick mixing directly at the print head is possible, as shown by Zhang et al. [8], but results in inferior mechanical properties of hardened material. Therefore, a vast majority of 3DCP solutions include a form of a non-pressurized material buffer tank (often called a hopper) attached directly at the print head. Inside that reservoir, printing mortar is maintained at the desired level in order to balance and resolve any inconsistencies in input material supply [77]. Additionally, a hopper provides more uniform operation of a pumping unit, enabling a proper duty cycle for extended service life of the system. Depending on the contained material volume, this intermediate stage can be equipped with a simple mixing device in order to mitigate any effects of early mortar setting. By constantly shearing the material, negative phenomena such as false set can be avoided.

A few solutions can be distinguished by the type of imposing mortar transfer through a nozzle. Most basic solutions are based on linear advancement of a piston-type mechanism deploying 3D concrete onto a print surface. Due to its simple construction, piston extruders are useful for preliminary testing 3DCP mixes, especially for green state characteristics, extrudability and buildability evaluation, as stated in previous paragraphs. In the case of more advanced 3DCP systems (e.g., gantry-type printers, robotic applications [19,117]), achieving a high rate of material flow and maintaining consistent and uninterrupted operation is crucial. For continuous operation, a screw-type extruder is employed. Those comprise a geared motor coupled with a rotating spiral with appropriate bearing arrangement. A form of mixing vanes can be attached to the extruder shaft in order to mitigate mix separation and improve material feeding. For more dynamic and accurate control of an extruder, a servomotor can be exploited. A variety of construction parameters can be adjusted for the screw, including length-to-diameter ratio, helix pitch, or core diameter, depending on the assumed material composition, aggregate size or presence of dispersed reinforcement. Although there are some exceptions to this method, as presented by Lowke et al. [118], material deposition in the vast majority of 3DCP systems is performed in a vertical, top-down process orientation. This approach is maintained even in applications of material deposition systems making use of six-axis kinematics of an industrial robot. Therefore, hydrostatic pressure generated as a result of the own weight of mortar assists in extrusion, significantly reducing power demand for the actual screw extruder. Additionally, it allows for more accurate flow control with reduced flow inertia. By adjusting rheological properties of a mix, uncontrolled material leaking along the extrusion path can be virtually eliminated [91,119].

### 6.3. Extrusion Nozzles

**Figure 6:**
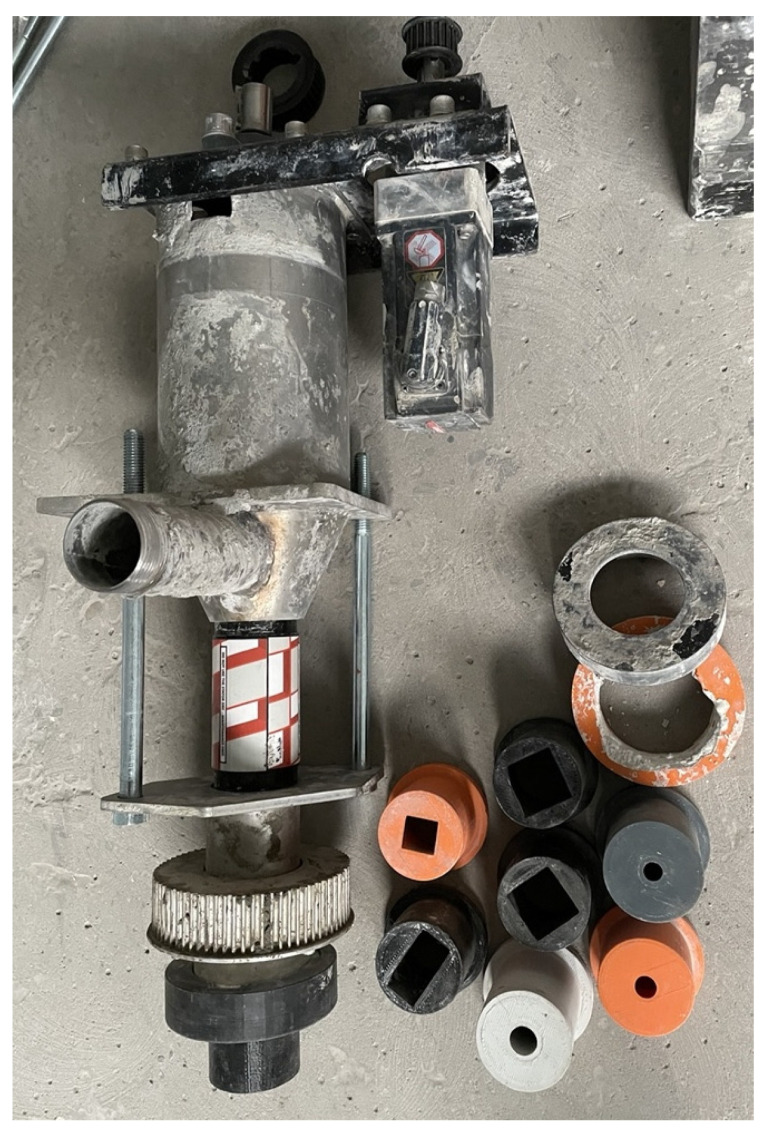
3DCP printhead with various orifice sizes and geometries (round 10, 15 mm, rectangular 20 by 40 mm, square 20 and 30 mm). Courtesy of ATMAT.

Depending on the specific 3DCP application and production scale, various nozzle geometries (as demonstrated by Figure 6) and sizes can be employed [17,74]. As the nozzle directly governs the shape fidelity and surface quality of the extruded filament, its geometry is a decisive factor for printing performance [84,120]. In large-scale applications, such as 3D-printed housing [121,122], rectangular or square nozzles are frequently used [123], as their increased cross-sectional area enables higher material output. Extruding mortar in a rectangular profile aligns naturally with the long, straight paths of wall structures [121]. By adjusting the ratio of extrusion width to height, both the target wall thickness and the required volumetric flow rate can be controlled. A consistent extrusion thickness in the direction of printing has been shown to improve layer support and vertical stability, particularly for increased layer heights [35,47]. Moreover, correlating extrusion width with output design reduces the number of required parallel passes, thereby decreasing total printing time and lowering costs. These advantages, however, come at the expense of greater mechanical complexity, as rectangular nozzles often require a rotary alignment mechanism to remain tangent to the print path [124]. A known drawback is the uneven distribution of material in sharp corners, which can create voids and initiate crack propagation in the cured mortar. These effects accumulate with subsequent layers as stresses intensify [92,97].

Circular orifices are often preferred for smaller-scale or laboratory applications, where simplified control and greater geometric freedom outweigh minor deformations of the filament cross-section [125]. In this context, round nozzles with orifice diameters of 15 mm are commonly employed [9,126], offering a practical compromise between extrusion stability, structural fidelity, and sample size. For large-scale construction, rectangular nozzles with dimensions such as 40 × 100 mm have been reported [127], particularly when mixes include larger aggregates and require higher throughput. Interestingly, extrusion nozzles fabricated using polymer-based additive processes such as Fused Filament Fabrication (FFF) [105] have also been adopted. These 3D-printed nozzles combine low production cost with customizable internal features, sufficient structural strength for extrusion, and the advantage of easy replacement in the event of wear.

### 6.4. Nozzle Additives

**Figure 7:**
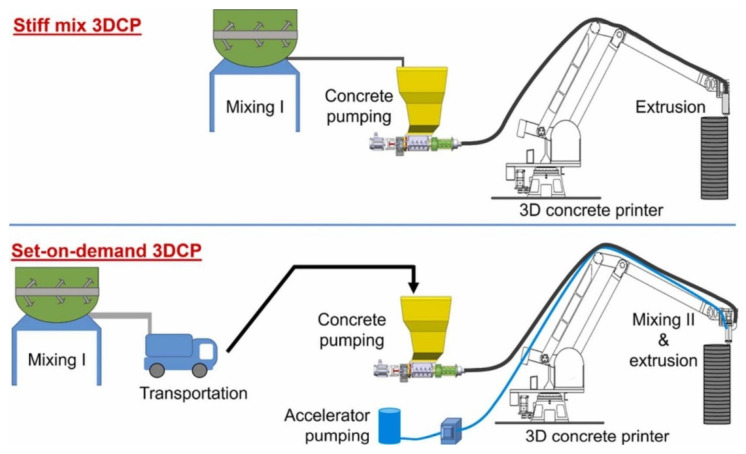
Alternative 3DCP system with accelerator injection at the printing nozzle proposed by Rehman et al. [129].

In 3DCP, the integration of nozzle additives and real-time injection techniques is a path in advancing process control, enhancing material properties, and achieving functional customization. Additives such as accelerators (e.g., calcium-based: CaCl_2_, Ca(NO_3_)_2_, sodium-based: Na_2_SiO_3_, NaAlO_2_) are commonly employed in set-on-demand strategies [128,129], where the concrete mix remains flowable during pumping but undergoes rapid stiffening near the nozzle transition zone (shown in Figure 7). The injection must be precisely controlled and restricted to extrusion nozzle proximity, in order to avoid premature material setting. This approach improves buildability and leads to a potential increase in process speed. However, challenges arise in ensuring accurate dosing to prevent premature setting within the delivery system, or conversely, inadequate hardening after extrusion, which may result in significant degradation of material mechanical properties [56,130]. Incorporating closed-loop control systems capable of real-time monitoring and adjustment of dosing parameters appears essential to mitigate these risks and ensure consistent performance.

Innovative techniques such as the direct injection of foaming agents and coloring pigments into the nozzle have expanded the functional and aesthetic possibilities of 3DCP. Injecting foaming agents facilitates the production of lightweight structures by controlling porosity, while on-demand pigmentation enables customization of concrete visual attributes. These methods, however, require precise calibration to achieve uniform mixing within the extrusion zone, prevent phase separation, and maintain structural integrity. While these advancements enhance the versatility of 3DCP, challenges such as uniform dispersion of mortar ingredients, consistent extrusion rates, and equipment compatibility must be addressed to ensure reliable outcomes.

### 6.5. Sensors

Equipping any 3DCP system with sensors for gathering both live and archived data can significantly contribute to enhanced performance and troubleshooting. Data acquisition can be incorporated into multiple stages of the process. Monitoring moisture levels of stored base materials helps to maintain consistency for mix composition despite varying environmental conditions, such as ambient temperature and weather conditions on the worksite. This contributes to maintaining an optimal water-to-binder ratio within the 3DCP mortar, which is essential for proper curing time duration and obtaining final mechanical properties of printed structures. Similarly, several studies show advantages arising from investigating the heat of hydration for a mortar [82]. While being relatively complicated for on-site applications, this method appears as a convenient tool for 3DCP mortar development in a more controlled environment. On the other hand, even the simplest feedback, such as of the current water supply temperature, may be found useful when troubleshooting in an actual worksite condition, especially in cases of continuous mix formulation systems. Any attempts at automating 3DCP mix formulation require the introduction of weighing or dosing units for all of the ingredients. Depending on the system scale and used ratios of a given material, a silo suspended on a set of load cells appears to be sufficient and a cost-effective means of monitoring remaining supply and controlling a dosing device (i.e., a feed screw).

For the final stage of the 3DCP workflow, material deposition, several application cases can be listed [131]. The implementation of machine vision (MV) and other sensor-based systems [132,133,134] for monitoring extrusion width, layer geometry, and defects offer unique data sources without disturbing a construction process in any way. Remarkably, image acquisition can be implemented into a real-time control system [132,135,136]. Despite the high cost of implementation, machine vision can significantly contribute to the development of more reliable printer machines in the future.

Machine vision research provides concrete examples of models applicable to 3DCP monitoring. EfficientNet, described by Kabir et al. [137], has been applied to automatically detect water levels in prismatic samples to estimate sorptivity, demonstrating how advanced architectures can support more automated and effective quality control. Similarly, the ResNet architecture [138], originally developed for generalized error detection and correction in 3D printing processes, could be adapted to extrusion-based cementitious printing to enhance defect recognition and improve process reliability.

Direct measurements include pressure transducers attached directly before an extrusion nozzle of a print head. Abrupt changes in nozzle pressure are the most direct way of detecting nozzle obstructions and following extrusion discontinuities (tearing, clogging [21,120,123]). However, observed variations such as pressure fluctuations due to pump pulsation or material heterogeneity require a more nuanced approach with sensor data filtering and trend analysis to correctly interpret signals. When included in a closed-loop machine control, such information can be exploited to account for extrusion rate corrections [119]. This approach excels for some more demanding print geometries (i.e., sharp corners, rapid accelerations). Finally, it is necessary to mention so-called indirect monitoring solutions. One of the simplest to be applied is monitoring the load on driving and feeding motors. It can be introduced by means of already used hardware in the case of servo or inverter drive units. Although gathered load values provide only approximate information on process consistency, they allow for detection of subsequent component wear and can potentially avoid catastrophic damage by triggering an overload alarm.

## 7. Conclusions

After covering all of the five processing stages, the following key findings can be presented as:

1. Established pumping and mixing solutions derived from the construction industry, such as progressive cavity pumps and high-shear mixers, are widely adapted for 3DCP purposes. Modifying and optimizing proven equipment is often more practical and economically viable than investing in system development through base research and prototyping.
2. Evaluation of mortar properties, including flowability, buildability, and extrudability is essential for achieving a reliable printing process. Some standardized testing methods from conventional concrete construction can be adapted for 3DCP. However, complementary methods are required to capture extrusion-specific behavior of the material. The task of achieving a suitable mix is a balance of pre- and post-deposition properties.
3. The implementation of closed-loop systems with real-time sensor feedback for precise process control applies well to 3DCP additive manufacturing. This strategy improves extrusion stability, reduces failure frequency, and supports the fabrication at larger scale and increased complexity.
4. Continuous monitoring of material-related properties and process parameters during mixing and printing enables early detection of faults. The integration of sensors and automated monitoring solutions enhances overall process reliability and directly contributes to the reduction of waste material.
5. The introduction of Recycled Concrete Aggregates as partial replacements for virgin materials often proves to be both sustainable and cost-effective strategy. However, it must be noted that their application requires quality control due to inherently increased variability of RCA properties and should always be considered with respect to the potential benefits.
6. The incorporation of supplementary cementitious materials contributes to a reduction in the environmental impact of concrete production by lowering the content of the greatest source of its carbon footprint—Virgin OPC. The SCMs can improve material processing characteristics, particularly pumpability and workability, depending on the scale of their integration into 3DCP mortars.

## 8. Future Development

### 8.1. Scaling Up

Adopting 3DCP for large-scale printing is widely recognized as a central challenge and focus area for the technology’s development [87]. The prospect of advancing the construction industry through faster, more efficient, and more sustainable practices represents the ultimate test of 3DCP’s maturity. Recent demonstrations have shown the feasibility of scaling the process to multi-story buildings and infrastructure projects, including bridges [139] and shelters. Nevertheless, scalability introduces technical demands such as robust equipment for continuous material delivery, precise layer deposition over extended time frames [86,117], and printing materials tailored to withstand scale effects. Parameters such as interlayer adhesion, extrusion stability, and curing kinetics become increasingly critical [53,88,140,141]. Compatibility with established construction standards and the process of securing regulatory approvals remain additional barriers to widespread adoption, as emphasized by Kreiger et al. [142].

Scalability also depends on the development of unified and quantifiable productivity metrics, such as output expressed in m^2^ per hour or downtime percentages. At present, the pursuit of such measures is limited, as most research prioritizes reliability and geometric accuracy over maximization of industrial-scale printing speed [143]. According to Ambily [110], scalability remains one of the most fundamental obstacles for transitioning 3DCP from niche applications to mainstream construction. Innovations in equipment, including multi-axis robotic arms and mobile printing systems [19], coupled with advancements in material formulation and delivery units, support increasing automation. Furthermore, the integration of real-time monitoring and closed-loop control mechanisms is expected to enhance process stability and ultimately enable scalable, standardized deployment of 3DCP.

### 8.2. Dynamic Mix Formulation

The significant complexity of 3DCP mortar formulation and higher process control requirements result in higher cost per material volume when compared to traditional cement-based mortars. However, since the material deposition process can be accurately controlled and adjusted, it is possible to balance this inherent drawback. In order to reduce the total volume of required material, structures may be optimized in terms of their topology regarding geometry best suited for mechanical properties. With the extrusion-based method, arcs, domes, and other means of stress-reducing features can be implemented, often reducing construction process time and enhancing structural reliability. Control over structure shell thickness, infill pattern, and its density is another aspect unique to 3DCP. When combined with advanced mixing units, those features allow for dynamic changes in mix composition. Several directions for development can be derived from such a combination. Firstly, with careful analysis, a structure being printed can be divided into primary (i.e., load bearing, overhangs, aesthetic as identified by Ting et al. [144]) sections of strategic viability and others fulfilling less demanding tasks such as internal wall patterns. Such an approach allows for application of a mortar with properties accurate to its future function. The function-based method can incorporate recycled aggregates and filler materials as a significant portion of mortar composition. Reduction of virgin binding material and introduction of lightweight filler ingredients (i.e., cork [46], expanded perlite [145], or shredded plastic waste [146,147]) may significantly decrease the cost per volume of 3D mixture, while greatly contributing to environmental impact of the whole construction process. However, this dynamic mix formulation idea comes with several inherent flaws already visible from the very concept stage. Unavoidable increase in complexity of the mixing system and expected difficulties in controlling extrusion arising from dynamically altering mortar properties are only a few to be listed from theoretical studies [3,5,85,148]. Summing up, while enhancing mixing units with capabilities to alter the mix composition during printing appears to be beneficial over many aspects such as cost reduction, improved performance, or environmental impact, it must be balanced with potential cost of development and increased process complexity. Still, the described approach appears as a valuable research direction in 3DCP development.

### 8.3. Portable Systems and Remote Construction Zones

Development directed towards modularity and portability is essential to create 3DCP systems capable of being relocated within a large-scale workspace. Portable systems are designed to be easily transported and deployed at different construction sites, enabling on-site printing of structures without the need to transport prefabricated components [18]. This strategy offers several advantages, including increased mobility, reduced logistical costs, shortened project timelines, and the feasibility of 3DCP construction in remote or hard-to-access areas. They are particularly beneficial in emergency scenarios, such as disaster recovery efforts, where they can be utilized to construct temporary shelters or rebuild infrastructure efficiently. A notable example of a portable 3DCP system is the technology developed by Xu et al. [19], which facilitates the on-site printing of structural components using a self-contained and automated approach. Nevertheless, portable systems face several challenges, such as harsh environmental conditions typical of construction sites, requirements for compact and lightweight design to ease transportation, and limited possibilities for on-site maintenance.

In parallel with addressing mobility, it is equally important to recognize the long-term impact of environmental exposure including moisture ingress, temperature fluctuations, and chemical attack on the durability of 3DCP structures.

Zhang et al. [33] reported that reduced incorporation of supplementary cementitious materials adversely affects frost resistance and chloride ingress, with the pore network particularly at interlayer interfaces being a decisive factor in permeability. Moreover, the relatively low water-to-cement ratios commonly applied in printable mixtures increase the susceptibility to autogenous shrinkage cracking [134]. Considering the layer-wise fabrication process and the presence of potentially weak planes at interlayer boundaries, repeated freeze–thaw cycles present a pronounced challenge, as they may intensify internal stresses and accelerate structural degradation [85].

## Figures and Tables

**Table 1:** Properties of various binding materials as presented in literature.

Material	Median Particle Size [μm]	Apparent Density [g/cm^3^]	Specific Surface Area [m^2^/kg]	Source
Ordinary Portland Cement	14	3.25	381	[32,33,34]
Fly Ash	53	2.55	454	[34]
Ground Granulated Blast Furnace Slag	1.95	2.90	1600	[30]
Silica Fume	16.5	2.21	22,315	[4,34,35]
Metakaolin	0.08	2.60	20,000	[34]

**Table 2:** Mortar evaluation methods applicable to 3DCP systems.

Parameter	Test Method	Equipment	Standard	Description	Adaptation for 3DCP
Workability/shape stability	Flow table test, slump test [16,46,105]	Flow table with vibratory actuation, mortar cone, tamper, measuring scale	EN 12350-2 [106], EN 1015-3 [107]	Measures deformation of a mortar upon form release.	Compares flow-related properties for mortars, can be limited in use [103].
Rheological properties	Concrete rheometer test [108]	Rotational Rheometer	ASTM C1749 [109]	Investigates plastic limit, viscosity and thixotropy.	Method designed for cement pastes, adaptation must consider presence of aggregates, requires modified testing geometries [110].
Green strength	Uniaxial compression test, penetration tests [94,111]	Compression testing machine, penetrometer	-	Measures initial resistance to compressive loading, which partially represents buildability.	Suitable for 3DCP.
Buildability	Layer setting test, layer stackability [78,82,103]	Material feeding device with extrusion nozzle, measuring scale	-	Measurement of structure wall deformation and sagging due to loading by vertical layers.	Applicable to 3DCP. Direct evaluation of structural stability.
Extrudability	Extrusion tests [36,80,103,112]	Ram extruder of controlled progression, print head with extrusion nozzle	-	Testing mortar ability for consistent nozzle flow. Visual examination of extruded material quality. Measurement of a force required to extrude, material deposition efficiency.	Suitable for 3DCP.
Open time	Early and finished curing formation time, consistency [36]	Vicat apparatus with penetrators and scale	EN 196-9 [113]	Measurement of sample penetration value (depth) taken in regular time intervals.	Can be adapted to estimate open time for a mortar, rather than curing time. May require adjustments in instruments.
Pumpability	Tribometer testing, rheometry [98,103,114]	Tribometer, sliding pipe rheometer	-	Testing material flows through a feed hose.	Suitable for 3DCP.
Structural build-up	Vane shear test, hydration heat measurement [92,115]	Vane rheometer, calorimeter	-	Evaluation of strength development over time and rate.	Applicable with limitations in equipment capabilities (rheometers).
Printability window	Print quality test, Extrusion limit test [100]	Laboratory-scale printer with a nozzle and mortar feeding device	-	Estimation of degradation of extrudability and buildability over time elapsed from mix formulation.	Direct representation of the 3DCP process.

## Data Availability

No new data were created or analyzed in this study. Data sharing is not applicable to this article.
